# Paraneoplastic Hypercholesterolemia Identified in an Adult Male Diagnosed With Metastatic Yolk Sac Tumor

**DOI:** 10.7759/cureus.44442

**Published:** 2023-08-31

**Authors:** Emeka P Agudile, Marina Khan, Puay Eng Tan, Olga Kozyreva

**Affiliations:** 1 Department of Internal Medicine, Steward Carney Hospital, Boston, USA; 2 Department of Social and Behavioral Sciences, Harvard T.H. Chan School of Public Health, Boston, USA; 3 Department of Internal Medicine, Steward Carney Hospital, Dorchester, USA; 4 Department of Pathology, Steward Carney Hospital, Dorchester, USA; 5 Department of Medical Oncology, Dana-Farber Cancer Institute, Brighton, USA

**Keywords:** germ cell tumours (gct), hepatoid, yolk sac tumor, hypercholesterolemia, paraneoplastic syndromes

## Abstract

A few cases of paraneoplastic hypercholesterolemia have been reported in patients with primary or metastatic liver cancer. We report a case of paraneoplastic hypercholesterolemia in a patient with a metastatic yolk sack tumor. The patient was a 52-year-old man who presented with abdominal pain, nausea, and vomiting. A computed tomography (CT) scan demonstrated massive hepatomegaly with innumerable large ill-defined hypo-densities and innumerable pulmonary nodules. Blood work demonstrated elevated bilirubin to 3.1 mg/dL, aspartate aminotransferase (AST) to 384 U/L, alanine aminotransferase (ALT) to 126 U/L, gamma-glutamyl transferase (GGT) to 574 U/L, lipase to 100 U/L, low-density lipoprotein (LDL) of 579 mg/dL, and cholesterol of >800 mg/dL. Tumor markers revealed alpha-fetoprotein (AFP) was 24,760 ng/mL, carcinoembryonic antigen (CEA) was 1.9 ng/mL, and cancer antigen 19-9 (CA19-9) was 86 U/mL. The tumor makers were obtained during the initial stages of the patient’s evaluation to help us narrow down the possible primary - focusing on the gastrointestinal tract and the pancreas. Although tumor markers are rarely of use in the early diagnosis of cancer due to their limited sensitivity and specificity; however, they can help diagnose the origin of cancer in patients presenting with advanced widespread disease such as our patient. Histopathology of his liver lesion biopsy demonstrated a metastatic yolk sac tumor (YST) with hepatoid differentiation. Since the patient succumbed rapidly, the primary tumor could not be ascertained, although the lack of a classic pattern for testicular tumor retroperitoneal lymphadenopathy makes extragonadal YST more likely. YSTs are major histologic subtypes of germ cell tumors (GCTs), and most frequently arise in the gonads. However, extragonadal GCT is sometimes seen and comprises about 2-5% of all GCTs in adult males aged 15-35 years. Extra gonadal GCT has been hypothesized to occur through aberrant migration of primordial germ cells or reverse migration of transformed germ cells from the testes, and persistence of pluripotent cells outside the gonads. Paraneoplastic syndromes associated with GCTs are rare. The pathophysiology of paraneoplastic hypercholesterolemia is hypothesized to involve the dysregulation of LDL receptors. Cancer-mediated mutations in the LDL receptor gene result in an abnormal LDL receptor, leading to autonomous cholesterol production by neoplastic cells. Also, tumor-secreted proprotein convertase subtilisin/kexin type 9 (PCSK9) has been implicated in the causation of paraneoplastic hypercholesterolemia. PCSK9 binds to and degrades the receptor for LDL particles on cell membranes. YST in adults is exceedingly rare. Paraneoplastic hypercholesterinemia is a very rare phenomenon reported in different cancers and we report the first case associated with YST.

## Introduction

A few cases of paraneoplastic hypercholesterolemia have been reported in patients with hepatocellular carcinoma (HCC), at the same time, some cases have been occasionally reported in other malignant diseases including renal cell carcinoma, lymphoma, and breast cancer [1-3]. Other groups of rare neoplasms with morphological and immunohistochemical features resembling HCC (hepatoid carcinomas) such as gastric hepatoid adenocarcinomas have been associated with elevated cholesterol levels [2,4].

Yolk sac tumors (YSTs) are histologic subtypes of germ cell tumors (GCTs), and most frequently arise in the gonads, but sometimes may arise in extragonadal sites [5]. Gonadal and extragonadal GCTs share some common features including occurrence in young men, and liver metastasis, where it can mimic other primary or metastatic liver malignancies associated with elevated alpha-fetoprotein (AFP) [6].

We report a case of paraneoplastic hypercholesterolemia in a patient with metastatic YST of unknown primary.

## Case presentation

We present a case of paraneoplastic hypercholesterolemia in a patient with metastatic YST to the liver and the lungs with an unknown primary.

The patient was a 52-year-old man with coronary artery disease (CAD), hyperlipidemia (HLD), hypertension (HTN), type 2 diabetes mellitus (DM), and hypogonadism on testosterone replacement for many years and presented with abdominal pain, nausea, and vomiting. The patient presented with progressively worsening right upper quadrant abdominal pain of about 2-3 months duration. He described his pain as a sharp pain that worsens with movement. His abdominal pain was associated with nausea, vomiting, and diarrhea. He also endorsed abdominal distention, especially after meals which usually improve with bowel movement. He also endorsed a weight loss of about 30 pounds during the last two months prior to his presentation to the hospital. He denied chest pain, shortness of breath, fever, chills, swelling in his neck or groin. At presentation, his abdomen was distended and tender to palpation especially on the right upper quadrant with hepatomegaly but normal bowel sounds. Other physical examination findings were unremarkable.

Our review of the patient’s charts showed that his lipid profiles have been well controlled and maintained within normal limits with a combination of atorvastatin and ezetimibe. It was documented that the patient was compliant with medication adherence. Table 1 shows the trends in the patient’s lipid profile two years prior to his last admission to the hospital with YST. The trends showed that all lipid parameters were within normal limits prior to his presentation, with dramatic upward trends in all the values subsequently (Table 1). At the same time, the patient’s DM was well controlled with a combination of metformin, dulaglutide, and regular insulin. His hemoglobin A1c ranged between 6.6% and 8.0%.

**Table 1:** The patient’s lipid profile trends during the course of his last hospital admission and two years prior to the last admission. LDL: low-density lipoprotein

Date Collected	Cholesterol	LDL	Triglyceride
08/10/2021	>800 mg/dL	579 mg/dL	664 mg/dL
06/14/2021	437 mg/dL	350 mg/dL	343 mg/dL
06/12/2021	396 mg/dL	305 mg/dL	342 mg/dL
03/26/2019	123 mg/dL	48 mg/dL	277 mg/dL

**Figure 1:**
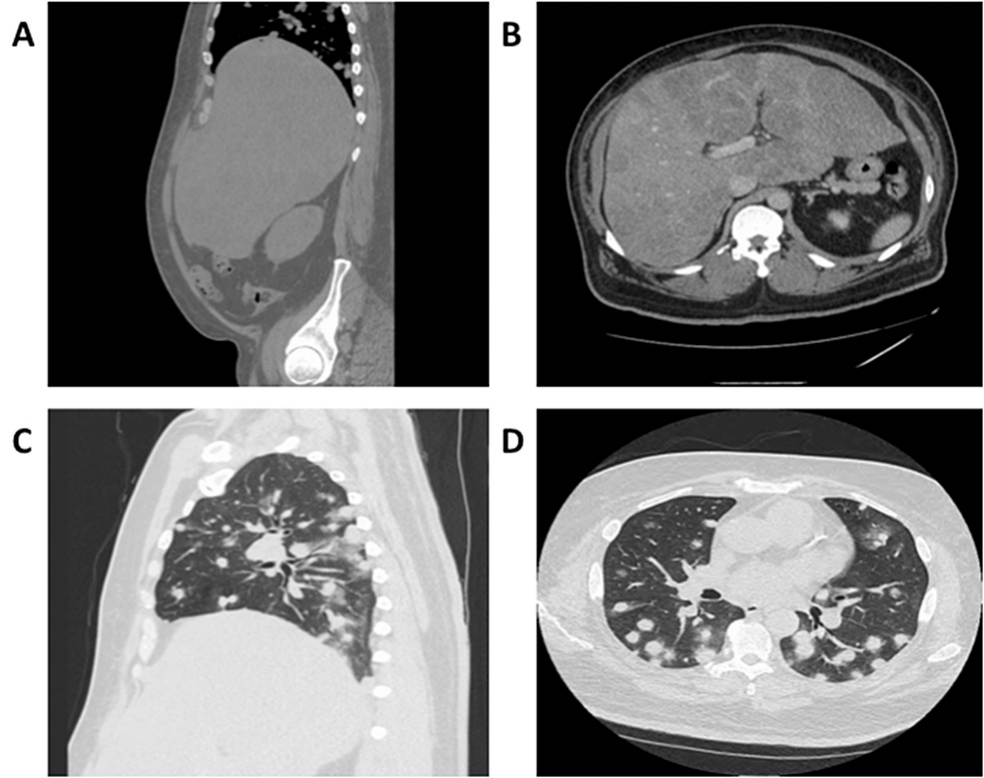
Abdomen/pelvis CT Scan: (A) sagittal and (B) transverse sections showing massive hepatomegaly with innumerable large ill-defined hypo-densities. Chest CT scan: (C) sagittal and (D) transverse sections showing innumerable pulmonary nodules. CT: computed tomography

A computed tomography (CT) scan demonstrated massive hepatomegaly with innumerable large ill-defined hypo-densities and innumerable pulmonary nodules (Figure 1). Blood work demonstrated elevated bilirubin to 3.1 mg/dL, aspartate aminotransferase (AST) to 384 U/L, alanine aminotransferase (ALT) to 126 U/L, gamma-glutamyl transferase (GGT) to 574 U/L, lipase to 100 U/L, low-density lipoprotein (LDL) of 579 mg/dL, and cholesterol of >800 mg/dL. Tumor markers revealed AFP was 24,760 ng/mL, carcinoembryonic antigen (CEA) was 1.9 ng/mL, and cancer antigen 19-9 (CA19-9) was 86 U/mL. Lipid panel one year ago was maintained within normal within the normal range on 80 mg of atorvastatin (cholesterol was 123 mg/dL, and LDL was 48mg/dL one year ago).

**Figure 2:**
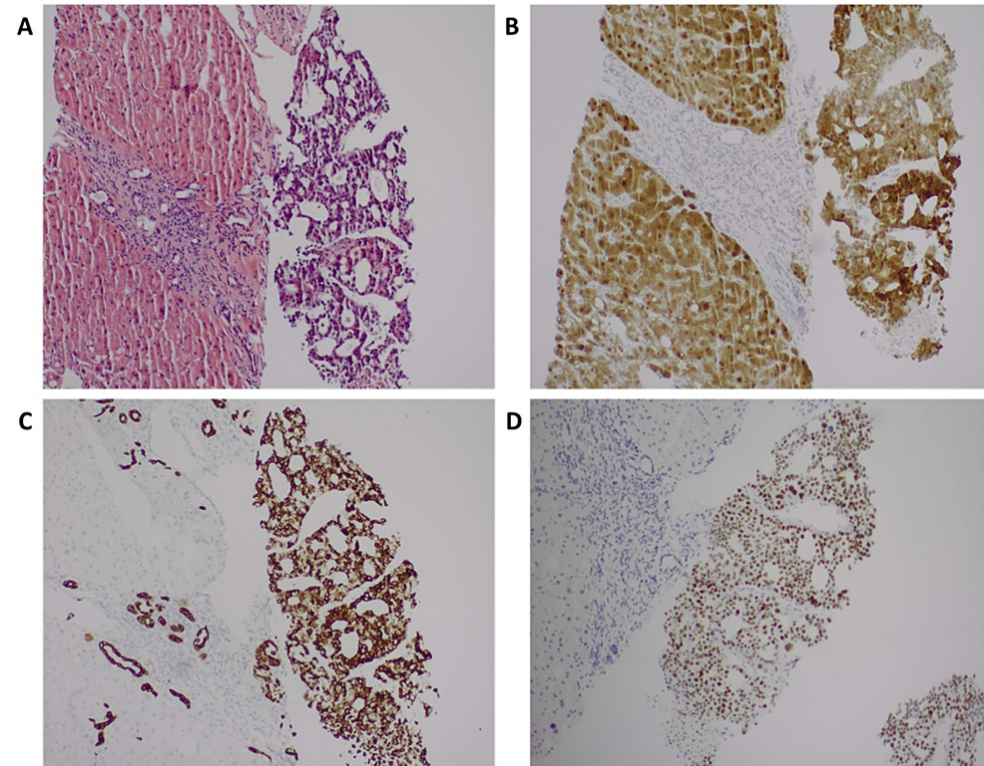
Histopathology examination (A) tumor nodule (right) and non-neoplastic liver (left) H&E 100x. (B) Tumor shares immunohistochemistry markers with liver cells (Hepar 1, bile canaliculi markers and Arginine 1, as shown here) Arginine IHC 100x. (C) Positive CK19 IHC in tumor, compared to liver cells (note positive internal control in bile ducts in liver), rendering a diagnosis of hepatocellular carcinoma unlikely. Cytokeratin 19 IHC 100x. (D) SALL4, a germ cell marker, is positive in tumor (positive nuclear stain), IHC 100X. This, together with positive staining for CDX2, CK19, and villin, supports a diagnosis of hepatoid yolk sac tumor metastasis. H&E: hematoxylin and eosin; Hepar1: anti-hepatocyte specific antigen; IHC: immunohistochemistry; CK19: cytokeratin-19; SALL4: Sal-like protein 4

Histopathologic examination of his liver lesion biopsy demonstrated a metastatic YST with hepatoid differentiation (hepYST). Immune-chemical stains of his liver lesion biopsy showed the tumor cells to be mucicarmine negative, cam5.2 positive, cytokeratin 7 negative, cytokeratin 19 positive, arginine 1 positive, hepar 1 positive, glypican 3 negative, polyclonal CEA positive canaliculi pattern, villin positive, sal-like protein 4 (SALL4) positive, caudal-type homeobox transcription factor 2 (CDX2) positive, CD10 (neutral endopeptidase) positive canaliculi pattern, CD56 (neural cell adhesion molecule) negative (Figure 2). Since the patient succumbed rapidly, the primary tumor could not be ascertained, although the lack of classic for testicular tumor retroperitoneal lymphadenopathy makes extragonadal YST more likely.

## Discussion

YSTs are major histologic subtypes of GCTs, and most frequently arise in the gonads. However, extragonadal GCT is sometimes seen and comprises about 2-5% of all GCTs in adult males aged 15-35 years [5]. The most common extragonadal locations include the mediastinum, the retroperitoneum, and the pineal gland. Primary YSTs of the liver are extremely rare [5].

Gonadal and extragonadal GCTs share some common features including occurrence in young men, metastasis to the liver, lung, and bones, presence of 12p karyotype abnormality, and sensitivity to cisplatin-based chemotherapy [6]. The presence of yolk sac elements in GCTs is associated with poor prognosis, but pure YSTs are rare in adult males [5].

YSTs usually display heterogeneous histologic patterns including microcystic/reticular, macrocystic, solid, glandular-alveolar, endodermal sinus, papillary, myxomatous, polysicular vitelline, enteric, and hepatoid patterns [7]. Schiller-Duval bodies and intracellular and extracellular PAS-positive hyaline globules are the hallmarks of YSTs. Also, YSTs are positive for AFP, cytokeratin AE1/AE3 (CK AE1/AE3), Glypican-3, and SALL4 but are negative for epithelial membrane antigen (EMA) and cytokeratin 7 (CK7) [7]. YSTs are characteristically associated with very high levels of serum AFP. The hepatoid variant of YST (hepYST) is a rare and aggressive tumor with a predominant pattern resembling HCC. HepYST is traditionally considered to signify a dismal prognosis [5].

Extragonadal GCT has been hypothesized to occur through aberrant migration of primordial germ cells or reverse migration of transformed germ cells from the testes, and persistence of pluripotent cells outside the gonads [6].

Paraneoplastic syndromes associated with GCTs are rare. Paraneoplastic hyperthyroidism, limbic encephalitis, and autoimmune hemolytic anemia may occur due to the presence of GCTs and can lead to diagnostic challenges for providers [8].

The pathophysiology of paraneoplastic hypercholesterolemia is hypothesized to involve the dysregulation of LDL receptors. Cancer-mediated mutations in the LDL receptor gene result in an abnormal LDL receptor, leading to autonomous cholesterol production by neoplastic cells. The abnormal LDL receptor is unable to bind LDL leading to decreased LDL clearance; this leads to a lack of downregulation of the hydroxymethylglutaryl-CoA (HMG-CoA) reductase enzyme and results in loss of negative feedback inhibition of the enzyme [4,9].

Also, tumor-secreted proprotein convertase subtilisin/kexin type 9 (PCSK9) has been implicated in the causation of paraneoplastic hypercholesterolemia. PCSK9 binds to and degrades the receptor for LDL particles on cell membranes. The combination of reduced LDL breakdown and increased LDL production leads to very high levels of serum LDL and cholesterol [10,11].

The main modalities of treatment for YSTs include chemotherapy with bleomycin, etoposide- and cisplatin-based regimens, followed by surgical resection of residual tumor or radiation [12]. Predictors of poor survival in GCTs include the presence of metastasis, mediastinal primary, non-pulmonary metastasis, and elevated markers (AFP >10,000 ng/mL, lactate dehydrogenase (LDH)>10x upper limit of normal, beta-human chorionic gonadotropin (beta-HCG) >50,000 IU/L) [13]. HepYST is traditionally considered to signify a dismal prognosis and has also been reported to be associated with a higher risk of relapse following initial treatment [5].

## Conclusions

YST in adults is exceedingly rare. It can mimic other primary or metastatic liver malignancies associated with elevated AFP such as multifocal HCC, hepatoblastoma, metastatic hepatoid carcinoma, and primary yolk sac of the liver. Paraneoplastic hypercholesterinemia is a very rare phenomenon reported in different cancers and we report the first case associated with YST. HepYST is traditionally considered to signify a dismal prognosis and has also been reported to be associated with a higher risk of relapse following initial treatment. Finally, knowledge of the syndromic manifestations and unusual imaging findings associated with YST helps guide early diagnosis and prompt treatment.
